# Medical students’ and teachers’ perceptions of sexual misconduct in the student–teacher relationship

**DOI:** 10.1007/s40037-013-0091-y

**Published:** 2013-10-30

**Authors:** Hanke Dekker, Jos W. Snoek, Johanna Schönrock-Adema, Thys van der Molen, Janke Cohen-Schotanus

**Affiliations:** 1 Institute for Medical Education, University of Groningen and University Medical Center Groningen, A. Deusinglaan 1, FC40, 9713 AV Groningen, the Netherlands; 2 Center for Research and Innovation of Medical Education, University of Groningen and the University Medical Center, Groningen, the Netherlands; 3 Department of Primary Care, University of Groningen and University Medical Center, Groningen, the Netherlands

**Keywords:** Student–teacher relationship, Sexual harassment, Misconduct, Boundary issues, Unprofessional behaviour, Gender differences

## Abstract

Teachers are important role models for the development of professional behaviour of young trainee doctors. Unfortunately, sometimes they show unprofessional behaviour. To address misconduct in teaching, it is important to determine where the thresholds lie when it comes to inappropriate behaviours in student–teacher encounters. We explored to what extent students and teachers perceive certain behaviours as misconduct or as sexual harassment. We designed—with a reference group—five written vignettes describing inappropriate behaviours in the student–teacher relationship. Clinical students (*n* = 1,195) and faculty of eight different hospitals (*n* = 1,497) were invited to rate to what extent they perceived each vignette as misconduct or sexual harassment. Data were analyzed using *t* tests and Pearson’s correlations. In total 643 students (54 %) and 551 teachers (37 %) responded. All vignettes were consistently considered more as misconduct than as actual sexual harassment. At an individual level, respondents differed largely as to whether they perceived an incident as misconduct or sexual harassment. Comparison between groups showed that teachers’ and students’ perceptions on three vignettes differed significantly, although the direction differed. Male students were more lenient towards certain behaviours than female students. To conclude, perceptions of misconduct and sexual harassment are not univocal. We recommend making students and teachers aware that the boundaries of others may not be the same as their own.

## Introduction

Development of professional behaviour has become a major part of education in competence-based medical curricula [1, 2]. Teachers play a key role in teaching professional behaviour, as they are important role models for trainee young doctors [3]. Unfortunately, teachers sometimes show unprofessional behaviour. Especially in the clinical workplace, where clinical teachers ought to be role models, misconduct in the student–teacher relationship (including sexual harassment) appears to occur [4, 5]. This is a serious problem, all the more because students are dependent on their clinical teachers for feedback and assessment [6]. To be able to outline a strategy to deal with (sexual) misconduct, it is important to determine which behaviours in the student–teacher encounters are perceived as (sexual) misconduct. In this study, we analyzed to what extent students and teachers consider incidents as misconduct and sexual harassment in the student–teacher relationship.

Unprofessional behaviours can be grouped into three different levels of severity: (1) atypical of the standard student–teacher relationship, (2) crossing boundaries and (3) violating boundaries [4]. In this study, we focus on unprofessional behaviours with sexual overtones. Examples of such behaviours range from inappropriate comments, unwelcome attention, flirtatious or sexual remarks to too personal questions and physical contact [6, 7]. The extreme forms of this type of misconduct are called sexual harassment. Sexual harassment—which is alarmingly often reported by students, with prevalence rates ranging from 18 to 60 % [6, 8–10]—has been shown to have a negative impact on students’ well-being [11–14]. Students who had been sexually harassed indicated that they functioned less well as a consequence and reported lower self-esteem and self-confidence [12]. Victims of sexual harassment also reported a diminished interest in or enthusiasm for their studies [10]. Furthermore, harassed students felt more stressed and depressed, drank more alcohol and tended to be more suicidal than non-harassed students [15]. There are also indications that sexual harassment affects students’ speciality choices [16]. For instance, female students confronted with incidents of harassment during their surgical clerkship did not specialize in general surgery, despite their initial intention and interest. The high prevalence rate of sexual misconduct in the student–teacher relationship and its negative impact on students denotes the importance of addressing sexual misconduct.

Medical schools have increasingly acknowledged the need to address sexual harassment. Several measures have been taken to address this issue. Hospitals and medical schools have, for instance, emphasized the fact that their students and teachers belong to a profession that has high ethical standards on maintaining appropriate professional boundaries in both the doctor–patient and the teacher–student relationship. In addition, hospitals and medical schools have formulated definite policies on sexual harassment and implemented procedures for reporting incidents of sexual harassment, and they have informed students and teachers about these policies and procedures [5]. Despite the fact that several measures have been taken to address (sexual) misconduct, students do not optimally make use of the established mechanisms for reporting abuse [12]. Reasons for not doing so are that students consider themselves as belonging to the lowest level of the hierarchy and they fear retaliation from abusive faculty, which could jeopardize their residency plans [15]. Another reason for not reporting sexual harassment is that female students feel that they, perhaps, may have been oversensitive [17]. Apparently, the measures taken are not enough to attain wholesome student–teacher relationships. Addressing sexual harassment actively in medical school curricula is needed to teach students how to deal with sexual misconduct and how to form and keep up professional relationships [18–20].

Methods to address sexual misconduct actively in curricula may include interactive educational sessions, in which students can discuss harassing experiences together and with their teachers [18]. To make these educational activities effective, it is essential to examine whether students and teachers have similar views on what constitutes (sexual) misconduct or even sexual harassment. We noticed that our students and teachers easily agree on sexual harassment when it concerns extreme sexual misconduct like rape. However, during their small-group meetings, our students also mentioned incidents with a sexual overtone in the student–teacher relationship that did not concern extreme forms of sexual misconduct. While the students who brought these incidents up obviously considered them as sexual misconduct, remarkably, not all teachers and students did so. This observation puzzled us and we decided to investigate whether such differences in opinion only occur incidentally or whether students and their teachers differ structurally in their opinions about the severity of unprofessional behaviours. We wanted to gain more insight into the opinions of students and teachers in order to provide input for the educational sessions on this topic. The aim of this study was to explore to what extent students and teachers perceive certain inappropriate situations in the student–teacher encounters as sexual misconduct or—more severely—as sexual harassment.

We formulated the following research questions:

1. To what extent are incidents perceived as (1) misconduct and as (2) sexual harassment?
2. To what extent are individuals consistent in their opinions: do individuals have an overall sensitivity for misconduct or sexual harassment?

We were also interested in group and gender differences in views on incidents:

3. Are there differences of opinion between students and teachers?
4. Are there differences of opinion between male and female students?

We also investigated whether female students who have already experienced harassment before/during medical school differed in their perception about incidents from those who have not experienced previous harassment. Therefore our last question was:

5. Are there differences of opinion between harassed and non-harassed female students?

## Methods

### Context

This study was performed at the University Medical Center Groningen in the Netherlands. The medical curriculum consists of a Bachelor’s and a Master’s programme, each lasting 3 years. Clinical clerkships start in the first Master’s year at the University Medical Center Groningen. For the clerkships of the second Master’s year, students are allocated to 1 of 7 different teaching hospitals in the north-eastern part of the Netherlands. After having written a Master’s thesis during the third year, students complete their basic medical training with a 20-week clerkship during which they act as junior doctors under strict supervision.

In the first and second Master’s year, students have to attend small-group sessions every other week, in which they discuss—in a structured way—various situations they experienced during clerkships. During these sessions, the subject of ‘sexual harassment’ has often been brought up as a problematic situation.

### Vignettes

We designed five vignettes to provide the participants with the context and background of unprofessional behaviours with sexual overtones in the student–teacher relationship. Real-life experiences that clerks mentioned during their small-group sessions were used as a source for the written vignettes. To cover different types of harassing behaviour, we used the classification scheme of Witte et al. [21] to define the content of the five vignettes. In order to improve face and content validity of the vignettes, we used a reference group of five teachers and five students. The five vignettes, which were originally in Dutch, are presented in English:

1. Sexist remark (*Barbie doll*): In the hospital where you are completing your clerkships, it is commonly known that gynaecologist A addresses all female clerks as ‘Barbie doll’. You, a female clerk, are on his ward for the first time and indeed, you are also addressed by him as ‘Barbie doll’.
2. Embarrassing comment (*Sexual joke*): While you are waiting in the coffee room for an operation, you (a young clerk) overhear surgeon A while he tells an explicit sexual joke to surgeon B.
3. Sexual overture (*Eye contact and invitation*): During morning report you (male clerk) notice that a female paediatric resident is constantly looking for eye contact. Afterwards she invites you to dinner.
4. Stereotypical comment (*Menstruation*): You are a female internal medicine clerk and during your evaluation at the end of the day your supervisor mentions that you performed much better last week. He asks you whether your poor clinical performance today may be attributed to your menstruation.
5. Clerk as harasser (*Provocative clothes*): You, a rehabilitation specialist, are in your office in the hospital at the end of the day. A female clerk enters and takes a seat very close to you. Her white coat is unbuttoned and you notice that she is wearing provocative clothes. She wants to know what she has to do in order to get a higher mark. She needs this higher mark because she wants to pass with honours.

### Participants

All students registered as first-, second- or third-year Master’s students were included in our study (*n* = 1,195). All clinical teachers registered at the administrative offices of the University Medical Center Groningen and seven teaching hospitals were invited to participate (*n* = 1,497). With permission of the various hospital boards, we administered the electronic questionnaires among staff members and residents.

The participants were asked to judge each vignette in two ways: (1) to what extent they perceived the vignette as misconduct and (2) to what extent they considered it as overt sexual harassment. We defined misconduct as behaviour perceived as crossing a boundary and sexual harassment as behaviour violating a boundary [5]. They were asked to rate their opinions on a five-point scale (1 = not at all, 5 = very much). We also added questions about gender, age, profession and whether the participant had experienced one or more sexually harassing situations him/herself.

### Ethical statement

At the time this study was carried out, national practice in the Netherlands did not require ethical approval for educational studies and surveys. However, in this study we adhered to the following ethical principles. The researchers had no hierarchical relationship with the participants. Participation was voluntary and anonymous. No rewards were offered.

### Analysis

Descriptive statistics were used to analyze to what extent the vignettes were perceived as misconduct and as sexual harassment. Pearson’s correlations were used to analyze to what extent students and teachers were consistent in their opinions about the different vignettes. We calculated correlations between the opinions on misconduct and sexual harassment within and between vignettes.

A paired *t* test was used to determine differences between sexual harassment and misconduct. Differences between students and teachers, male and female students, and harassed and non-harassed female students were analyzed using the independent samples *t* test. Due to the high number of comparisons, we used Bonferroni correction, with the Bonferroni correction alpha being 0.05/35 and, therefore, interpreted differences with *p* ≤ 0.001 as significant. To indicate the importance of the differences, effect sizes (ES) were calculated using the formula described by Field [22]. Consequently, we applied ES = 0.10 (small effect); ES = 0.30 (medium effect) and ES = 0.50 (large effect).

## Results

### Descriptives

The questionnaire was completed by 643 students (response rate 54 %); 77 % were female and 23 % were male. The female/male ratio of the total student population is 70–30 %. The students’ average age was 23.8 years. The questionnaire was completed by 551 clinical teachers (response rate 37 %), of whom 36 % were female and 64 % male. The average age of the clinical teachers was 42.9 years. The respondents were 181 medical specialists of the University Medical Center Groningen (33 %), 138 medical specialists from teaching hospitals (25 %), 154 residents (28 %), 48 general practitioners (9 %), 18 public health doctors (3 %) and 12 persons with a different background (2 %). In the past, 130 clerks (20 % of all participating clerks) and 65 clinical teachers (12 % of all participating teachers) had experienced one or more sexually harassing incidents themselves. Of the 130 harassed clerks, 124 were females (95 %).

### Views on misconduct and sexual harassment

All vignettes were perceived significantly more often as misconduct than as sexual harassment (Table 1). Respondents rated the vignettes ‘Stereotypical comment’ and ‘Clerk as harasser’ as the most improper and the vignette ‘Embarrassing comment’ as the least improper. An in-depth exploration of individual ratings of the vignettes revealed a large variation in opinions. Every vignette was perceived by some respondents as very sexually harassing and as real misconduct, whereas others did not perceive them as sexually harassing or misconduct at all. For instance, the vignette ‘Embarrassing comment’—which was rated as least improper—was perceived by 9 % of the respondents as still highly sexually harassing behaviour.

**Table 1:** Views on sexual harassment and misconduct

*N* = 1,194	Low (%)	Neutral (%)	High (%)	Mean (SD)	*t* (df)	*p*	ES
Sexist remark							
Misconduct	20	20	60	3.52 (1.09)	35.85 (1,186)	0.000*	0.72
Sexual harassment	54	28	18	2.47 (1.06)			
Embarrassing comment							
Misconduct	52	25	23	2.54 (1.15)	22.38 (1,189)	0.000*	0.54
Sexual harassment	73	18	9	1.97 (1.01)			
Sexual overture							
Misconduct	44	25	31	2.75 (1.24)	16.97 (1,188)	0.000*	0.44
Sexual harassment	57	26	17	2.33 (1.14)			
Stereotypical comment							
Misconduct	5	10	85	4.31 (0.88)	39.82 (1,190)	0.000*	0.76
Sexual harassment	18	18	64	2.87 (1.25)			
Clerk as harasser							
Misconduct	5	8	87	4.25 (0.90)	21.39 (1,187)	0.000*	0.53
Sexual harassment	42	26	32	3.62 (1.17)			

Respondents rated the vignettes on both misconduct and sexual harassment on a scale from 1 = not at all to 5 = very much. Low = percentage of respondents scoring 1 or 2, neutral = percentage of respondents scoring 3, high = percentage of respondents scoring 4 or 5. * Significant at 0.001 level (differences between scores on misconduct and sexual harassment)

Effect size low = 0.10, medium = 0.30 and large = 0.50

### Relations between misconduct and sexual harassment scores, both within and between vignettes

Within the vignettes, the correlations between individual scores on misconduct and sexual harassment varied between 0.35 and 0.74 (Table 2, bold numbers). Between the vignettes, the correlations between individual scores on misconduct varied between 0.09 and 0.33 (Table 2, italic numbers) and the correlations between individual scores on sexual harassment varied between 0.20 and 0.46 (Table 2, underlined numbers).

**Table 2:** Relations between misconduct scores and sexually harassing scores, both within and between vignettes

*N* = 1,194	Sexist remark		Embarrassing comment		Sexual overture		Stereotypical comment		Clerk as harasser	
	Misc.	Sexu.	Misc.	Sexu.	Misc.	Sexu.	Misc.	Sexu.	Misc.	Sexu.
Sexist remark										
Misconduct	1.00	**0.56**	*0.33*	0.26	*0.09*	0.12	*0.26*	0.17	*0.22*	0.18
Sexual harassment		1.00	0.32	0.46	0.14	0.25	0.12	0.36	0.13	0.23
Embarrassing comment										
Misconduct			1.00	**0.68**	*0.25*	0.26	*0.13*	0.24	*0.18*	0.15
Sexual harassment				1.00	0.25	0.33	0.02	0.34	0.10	0.20
Sexual overture										
Misconduct					1.00	**0.74**	*0.15*	0.23	*0.17*	0.21
Sexual harassment						1.00	0.07	0.33	0.15	0.31
Stereotypical comment										
Misconduct							1.00	**0.35**	*0.30*	0.16
Sexual harassment								1.00	0.16	0.30
Clerk as harasser										
Misconduct									1.00	**0.55**
Sexual harassment										1.00

All correlations are significant at 0.01 level. Bold = correlations within vignettes between misconduct and sexual harassment, italic = correlations between vignettes concerning misconduct, underlined = correlations between vignettes concerning sexual harassment

### Group differences

Students’ and teachers’ opinions differed significantly on the vignettes ‘Sexist remark’, ‘Embarrassing comment’ and ‘Sexual overture’ for both misconduct and sexual harassment (Table 3). However, the direction differed. The students were more permissive towards the vignettes ‘Sexist remark’ and ‘Embarrassing comment’, whereas the teachers were more permissive towards ‘Sexual overture’.

**Table 3:** Students versus teachers

	Students (*n* = 643) Mean (SD)	Teachers (*n* = 551) Mean (SD)	*t* (df)	*p*	ES
Sexist remark					
Misconduct	3.33 (1.09)	3.73 (1.06)	−6.37 (1,170)	0.000*	0.18
Sexual harassment	2.27 (0.99)	2.70 (1.10)	−7.09 (1,109)	0.000*	0.21
Embarrassing comment					
Misconduct	2.38 (1.09)	2.73 (1.12)	−5.23 (1,122)	0.000*	0.15
Sexual harassment	1.83 (0.93)	2.13 (1.08)	−5.05 (1,089)	0.000*	0.15
Sexual overture					
Misconduct	2.92 (1.20)	2.56 (1.28)	4.92 (1,133)	0.000*	0.14
Sexual harassment	2.44 (1.12)	2.20 (1.15)	3.64 (1,187)	0.000*	0.11
Stereotypical comment					
Misconduct	4.26 (0.90)	4.37 (0.49)	−2.18 (1,189)	0.029	0.06
Sexual harassment	2.89 (1.26)	2.84 (1.25)	0.63 (1,190)	0.527	0.02
Clerk as harasser					
Misconduct	4.23 (0.90)	4.27 (0.90)	−0.83 (1,188)	0.406	0.02
Sexual harassment	3.68 (1.11)	3.55 (1.23)	1.88(1,112)	0.059	0.06

Effect size low = 0.10, medium = 0.30 and large = 0.50

* Significant at 0.001 level

Male students were more permissive on the vignettes ‘Clerk as harasser’, ‘Sexual overture’ and ‘Embarrassing comment’ (Table 4) than were female students.

**Table 4:** Male students versus female students

	Male (*n* = 150) Mean (SD)	Female (*n* = 492) Mean (SD)	*t* (df)	*p*	ES
Sexist remark					
Misconduct	3.24 (1.09)	3.36 (1.09)	−1.21 (637)	0.226	0.05
Sexual harassment	2.18 (0.96)	2.30 (0.99)	−1.24 (637)	0.214	0.05
Embarrassing comment					
Misconduct	1.97 (0.96)	2.50 (1.10)	−5.70 (278)	0.000*	0.32
Sexual harassment	1.59 (0.82)	1.90 (0.95)	−3.61 (637)	0.000*	0.14
Sexual overture					
Misconduct	2.33 (1.08)	3.10 (1.17)	−7.21 (638)	0.000*	0.27
Sexual harassment	1.87 (1.01)	2.61 (1.10)	−7.84 (270)	0.000*	0.43
Stereotypical comment					
Misconduct	4.01 (1.01)	4.34 (0.84)	−4.07 (638)	0.000*	0.16
Sexual harassment	2.67 (1.17)	2.95 (1.28)	−2.45 (639)	0.014	0.10
Clerk as harasser					
Misconduct	3.91 (1.09)	4.32 (0.80)	−4.25 (201)	0.000*	0.29
Sexual harassment	3.27 (1.26)	3.81 (1.03)	−4.82 (213)	0.000*	0.31

Effect size low = 0.10, medium = 0.30 and large = 0.50

* Significant at 0.001 level

We did not find any statistical differences between the opinions of harassed and non-harassed female students (Table 5).

**Table 5:** Harassed female students versus non-harassed female students

	Harassed (*n* = 124) Mean (SD)	Non-harassed (*n* = 368) Mean (SD)	*t* (df)	*p*	ES
Sexist remark					
Misconduct	3.40 (1.14)	3.35 (1.08)	0.41 (489)	0.597	0.02
Sexual harassment	2.30 (1.00)	2.30 (0.99)	0.01 (489)	0.909	0.00
Embarrassing comment					
Misconduct	2.67 (1.19)	2.45 (1.06)	1.84 (193)	0.067	0.13
Sexual harassment	1.98 (0.98)	1.88 (0.94)	0.98 (487)	0.330	0.04
Sexual overture					
Misconduct	3.15 (1.19)	3.08 (1.16)	0.52 (488)	0.806	0.02
Sexual harassment	2.64 (1.09)	2.60 (1.10)	0.34 (488)	0.720	0.02
Stereotypical comment					
Misconduct	4.37 (0.81)	4.33 (0.85)	0.43 (488)	0.495	0.02
Sexual harassment	3.06 (1.32)	2.92 (1.26)	1.04 (489)	0.370	0.05
Clerk as harasser					
Misconduct	4.24 (0.93)	4.35 (0.75)	−1.17 (181)	0.197	0.09
Sexual harassment	3.75 (1.04)	3.83 (1.02)	−0.77 (487)	0.756	0.03

Effect size low = 0.10, medium = 0.30 and large = 0.50

* Significant at 0.001 level

## Discussion

In this study, we explored medical students’ and teachers’ perceptions about written vignettes describing inappropriate student–teacher encounters. All vignettes were consistently considered more as misconduct than as actual sexual harassment. At the individual level, we found a large variation in perceptions of misconduct and sexual harassment. Both within and between respondents, opinions differed from incident to incident: some respondents perceived a particular incident as very harassing and the other incidents as not harassing at all, while other respondents, in contrast, perceived one of the other vignettes as particularly harassing. All incidents were considered by some as overt misconduct or as very sexually harassing and by others as not misconduct or sexual harassment at all. There was no consistency across respondents regarding which incidents they considered most serious or, in other words, a general sensitivity for sexual harassment does not seem to exist.

Upon comparison of the opinions of subgroups in our respondent sample, we found some differences between teacher and student perceptions, but there was no clear pattern. In some cases, teachers were more permissive, in other cases students were more lenient. This outcome differs from the results of a study conducted by Ogden et al. [23], who found that clinical teachers considered more behaviours to be abusive than did students. In addition, we found that male students were more lenient towards certain behaviours than female students. An explanation for the fact that females perceived vignettes more as sexual harassment and as misconduct than males did may be that female students themselves are more often victims of sexual harassment—in our study, for instance, 96 % of the harassed students were female—and that these experiences affect their perceptions of the vignettes [10, 15, 24, 25]. If this line of reasoning is true, we might expect different views between harassed and non-harassed students. However, we did not find any differences in the opinions of harassed and non-harassed female students.

A strength of our study is that we used real-life experiences of clerks to investigate their perceptions and those of their teachers. Considering each of these experiences was felt to be inappropriate by some and as acceptable by others stresses the need to address a range of examples of misconduct in education. In our study, the vignette in which the clerk was the harasser was perceived as most sexually harassing. Teachers indicated that they recognized the situation and that it made them feel uncomfortable and insecure because they doubted whether the clerk intentionally tried to harass or whether they had misunderstood the situation. Based on their study of sexual harassment of female doctors by patients, Schneider and Phillips [26] suggest that this phenomenon can be explained by the so-called contra power. Contra power harassers have a way of obstructing formal power in spite of an explicit power imbalance. They tend to use low-risk behaviour, because it can easily be explained as a misunderstanding. In our vignette, the student fulfilled the role of the harasser, although the teacher held the formal power. Considering our findings, we recommend not to limit the discussion of misconduct to stereotypical incidents in the student–teacher relationship in which the ‘harasser’ is an older male and the ‘victim’ is a young female, but also to discuss vignettes in which the clerk is the harasser.

We realize that our study, in particular the content of the vignettes, was limited to the Dutch context. Although our vignettes were based on real-life situations reported by our Dutch students, they may be culturally biased. The Netherlands is a country characterized by individualism and feminism and gender equity in social and sexual interaction is generally accepted in the Netherlands [27, 28]. Although the content of the vignettes may be specific to the Dutch culture—and maybe also to other countries high in individualism and femininity—the principle of incidents being differently valued by individuals, i.e. being considered as misconduct or not, may hold in different cultures. Future research is needed to find out whether our outcomes—that individuals differ strongly as to what they consider as acceptable or not acceptable—are also valid for other countries or cultures.

The most important finding of our study is the observation that students and their teachers differ structurally in their opinions about the severity of unprofessional behaviours. The differences in interpretations can be caused by many factors such as the respondent’s individual past experiences, personality, cultural background, religious background, family background or the way he/she was brought up. The thresholds for perceiving incidents as misconduct or even sexual harassment seem highly personal and which incidents are considered as most serious varies strongly across individuals. This outcome forms a plausible explanation for why it is so hard to define and address sexual harassment in medical schools. The lack of uniformity in answers hinders the formulation of strict guidelines on which conduct is permissible and which conduct is not.

The practical implications of these outcomes are that a different approach is required to address sexual misconduct. Interactive educational sessions in which students and teachers discuss vignettes together are recommended [18]. As study material for these educational sessions, we suggest—based on our outcomes—to present several (at least 4–5) different vignettes about the student–teacher relationship per session. Using vignettes with incidents of differing severity in a session helps to create awareness that there are individual differences in thresholds concerning what is acceptable and what is not. Such awareness may help participants of these educational sessions to realize and respect that other peoples’ boundaries may not be the same as their own. Second, we recommend taking gender differences into account in the educational sessions. Because male students were more permissive on some vignettes than female students, awareness of differences between males and females may be increased by composing mixed-gender small groups.

We would like to stress the importance of developing awareness among students and teachers, because it is not easy to find the right balance between closeness and distance. It is the teacher’s task to help students to acculturate or socialize in the ‘community of practice’ of the medical profession [18]. Therefore, the role of the clinical teacher requires a certain level of collegial and social closeness [18]. Since the student–teacher relationship is by definition one of unequal powers, however, finding the right balance between closeness and distance may be difficult [4]. Teachers may be unaware that they are deviating from professional standards of conduct. Because of power inequality, students may find it hard to negotiate boundaries or defend themselves against boundary crossings. The issue may be further complicated by the fact that what is acceptable is often not a clear-cut matter of right and wrong. Therefore, we encourage educators to explicitly pay attention to raising awareness of students and teachers that what is acceptable to some people may not be acceptable at all to others.

## Conclusion

Students’ and teachers’ perceptions of sexual harassment and misconduct are not univocal. Our findings indicate that students and teachers recognize the concept of misconduct sooner than sexual harassment. We suggest teaching both students and teachers to be aware of different situations of misconduct within their mutual relationships, and to realize and respect that other people’s boundaries may not be the same as their own.

## Essentials

- Students and teachers perceive the five vignettes more as misconduct than as sexual harassment.
- At an individual level, students and teachers vary greatly in their perceptions of misconduct and sexual harassment in their mutual relationships.
- At group level, male students perceive three of the five vignettes as more permissive than their female peers.
- A general sensitivity for sexual harassment does not seem to exist.
- Educational sessions in which students and teachers discuss vignettes together can help them to create awareness of professional boundaries in their mutual relationships.
